# Effects of Early Bilingual Experience with a Tone and a Non-Tone Language on Speech-Music Integration

**DOI:** 10.1371/journal.pone.0144225

**Published:** 2015-12-11

**Authors:** Salomi S. Asaridou, Peter Hagoort, James M. McQueen

**Affiliations:** 1 Max Planck Institute for Psycholinguistics, Nijmegen, The Netherlands; 2 Donders Institute for Brain, Cognition and Behaviour, Radboud University, Nijmegen, The Netherlands; 3 Behavioural Science Institute, Radboud University, Nijmegen, The Netherlands; UNLV, UNITED STATES

## Abstract

We investigated music and language processing in a group of early bilinguals who spoke a tone language and a non-tone language (Cantonese and Dutch). We assessed online speech-music processing interactions, that is, interactions that occur when speech and music are processed simultaneously in songs, with a speeded classification task. In this task, participants judged sung pseudowords either musically (based on the direction of the musical interval) or phonologically (based on the identity of the sung vowel). We also assessed longer-term effects of linguistic experience on musical ability, that is, the influence of extensive prior experience with language when processing music. These effects were assessed with a task in which participants had to learn to identify musical intervals and with four pitch-perception tasks. Our hypothesis was that due to their experience in two different languages using lexical versus intonational tone, the early Cantonese-Dutch bilinguals would outperform the Dutch control participants. In online processing, the Cantonese-Dutch bilinguals processed speech and music more holistically than controls. This effect seems to be driven by experience with a tone language, in which integration of segmental and pitch information is fundamental. Regarding longer-term effects of linguistic experience, we found no evidence for a bilingual advantage in either the music-interval learning task or the pitch-perception tasks. Together, these results suggest that being a Cantonese-Dutch bilingual does not have any measurable longer-term effects on pitch and music processing, but does have consequences for how speech and music are processed jointly.

## Introduction

Speech and music share fundamental building blocks: sounds. Despite the fact that sounds are organized in distinct representational systems for speech and music using different aspects of the acoustic signal [1], the cortical and subcortical mechanisms processing them seem to be overlapping[2–5]. It therefore comes as no surprise that transfer effects between speech and music arise, such that auditory expertise in music or in speech modulates processing in the other domain (for a review, see [6]). There is an asymmetry, however, in the number of studies on music-to-language versus language-to-music effects, with fewer of the latter type. The present study examines language-to-music effects in a novel way. Previous studies of this type have focused largely on tone-language speakers. The assumption is that such speakers have been “trained” in pitch, a sound property fundamental for music, albeit in a linguistic context. The question usually addressed is the following: Can tone-language speakers’ ability to process pitch in speech transfer to pitch processing in a non-linguistic or musical context? Here, we ask this question with respect to Cantonese-Dutch bilinguals, who may have particular auditory expertise in pitch processing because of their experience with pitch as a cue to multiple lexical and intonational distinctions.

A number of studies have found evidence that tone-language speakers are better on musical pitch tasks than non-tone-language speakers. Mandarin speakers outperform English speakers in detecting contour and interval changes in simple melodies [7]; Thai speakers are faster than English speakers in detecting contour characteristics in music stimuli[8]; and Cantonese speakers are better than nonmusician English speakers in skills such as melody discrimination and tonal memory [9,10]. Tone-language speakers are also superior in pitch discrimination tasks [9,11]and their advantage is not only evident in perception but also in the production of music intervals [12].

But tone-language experience can also induce limitations or biases to pitch perception. Mandarin speakers, for example, show biased responses when asked to identify non-linguistic pitch contours with small pitch excursions, identifying them more often as rising than as falling [13]. In accordance with that, Mandarin speakers are significantly worse than non-tone-language speakers in detecting downward pitch changes in simple melodies [14]. These limitations are caused by interference from language experience, given that rising tones in Mandarin consist of small frequency excursions while falling tones have large excursions and are therefore less finely tuned in Mandarin speakers [13,14].

There is cortical and subcortical electrophysiological data showing sharpened pitch encoding in tone-language speakers[2,11,15]. However, although precision in neuronal pitch encoding is necessary for perceptual advantages to occur, it is not sufficient. Bidelman, Gandour, and Krishnan [16] found that tone-language experience enhances subcortical pitch processing in a manner similar to musical experience. While English speaking musicians also showed better pitch perception alongside brainstem responses, tone-language speakers did not show such a behavioral advantage [16]. Another study comparing tone-language speakers to musicians [17] also failed to find a tone-language advantage in tone identification whereas it did find an advantage of musical training.

One potential source of this discrepancy between the effects of tone-language and music experience is the explicit knowledge musicians acquire through purposeful training. Explicit knowledge, such as a sophisticated vocabulary for different sound phenomena and concepts, allows musicians to develop a metacognitive understanding of sound. This is of importance for transfer effects to occur, as attentional mechanisms are involved in perceptual attunement to stimulus properties [18,19]. Music experience triggers attunement to auditory features through attentional exercises and purposeful repetition. These are both essential elements in music training [19] but are rather unnecessary in natural first language acquisition.

With this background in mind, we set out to investigate language-to-music transfer effects in individuals in whom metacognitive sound processing has been enhanced not through music training, but through linguistic experience: early bilinguals [20]. By ‘early bilinguals’ we refer to individuals who have been exposed to two languages from birth. Early bilinguals can be considered “auditory experts” since they outperform their peers in metalinguistic phonological skills [21,22]; that is, they have learned to reflect on language sounds and learn to dissociate, manipulate, and inhibit different phonetic systems from early infancy onwards.

Early experience with two languages induces plastic changes in the brain’s function [23]and structure[24]. One of the most interesting findings is that early bilinguals have greater grey and white matter density in the primary auditory cortex compared to late bilinguals [25]. This has been attributed to early exposure to different phonetic inventories. Early second language onset is thus an important cause of plastic changes in the human cortex.

What then are the consequences of such plastic changes in sound processing performance? Our hypothesis was that linguistic experience, that is, speaking two languages from an early age on, will influence sound processing outside the domain of language. We chose a bilingual population with experience in a tone language (Cantonese) and a non-tone language (Dutch), assuming that this is a circumstance under which pitch processing is put under the most pressure, due to the diversity in the use of pitch information in this case [26]. Dutch uses pitch to signal intonational distinctions at the sentence level and to signal lexical stress distinctions [27]. Cantonese also uses pitch for sentence-level intonation [28], but uses it especially to convey lexical meaning (e.g. to distinguish meanings in words differing only in tone). Moreover, Cantonese has a more complex tone repertoire than the more frequently studied Mandarin language. The six tones in Cantonese include both contour and level tones, and hence require fine-grained F0 processing abilities [29]. Learning to master control over such different phonetic inventories could potentially train Cantonese-Dutch bilinguals in a manner comparable to music training.

Whereas the majority of prior studies have focused on transfer effects, that is, the beneficial effect of past experience in the linguistic domain on current processing in the music domain, in this study we set out to test two other speech-music effects as well. The first is the online interaction that occurs when speech and music are processed simultaneously in sung speech. The goal was to investigate how experience with two different linguistic pitch systems influences this interaction. The second is whether there are long-term consequences of learning Cantonese and Dutch on the ability to learn new sounds. In particular, we wanted to test the Shared Sound Category Learning Mechanism (SSCLM) hypothesis put forward by Patel (2008)[1]. According to this hypothesis, music and speech may rely on the same learning mechanism, one which extracts spectrotemporal regularities in the acoustic signal and creates the respective sound categories. We wanted to test whether the efficacy of the SSCLM is increased in Cantonese-Dutch bilinguals, given the fact that it is put under higher pressure to perform during a sensitive period of language development.

We assessed online interactions using a speeded classification task introduced by Kolinsky, Lidji, Peretz, Besson, & Morais (2009) [30] and based on Garner interference [31]. In this task, participants listen to sung words and classify them according to a pre-specified dimension: musical or phonological. In the musical dimension participants judge the music interval as either ascending or descending, and in the phonological dimension the judge the identity of the vowel in the sung word. Kolinsky et al. found that music and vowels are processed integrally and thus participants cannot filter out irrelevant variation in one dimension when performing a judgment on the other. That is, they are faster when both dimensions vary consistently (e.g. ascending interval paired with vowel x) and slower when they vary inconsistently (e.g. all possible combinations between intervals and vowels). The ability to ignore irrelevant variation in the acoustic signal seems to play an important role in the acquisition of nonnative phonemes where perceptual interference from irrelevant dimensions from one’s native language can arise [32].

We had two alternative hypotheses with regards to this task. On the one hand, we expected that Cantonese-Dutch bilinguals will have mastered the ability, from early on in development, to switch between two language systems that make different uses of pitch information. In order to accomplish this, they would have to learn to ignore, for each of their languages, pitch variation that was irrelevant in that language (but relevant in the other language). According to this hypothesis, Cantonese-Dutch bilinguals should exhibit less interference than Dutch control participants when asked to perform the speeded classification task, because the task requires simultaneous evaluation of phonemic and pitch variation. On the other hand, it has been found that tone-language speakers show more interference when processing pitch and phonemes simultaneously[33,34]. Specifically, Lee and Nusbaum [33] found that, while performing a speeded classification task, Mandarin speakers showed interference from constant pitch information whereas English speakers did not. Due to their linguistic experience, tone-language speakers may have more highly-developed processing strategies, such that they integrate segmental and suprasegmental information more than non tone-language speakers do [33]. An alternative hypothesis would therefore be that, as speakers of a tone language, it may be harder for Cantonese-Dutch bilinguals to ignore pitch information compared to participants who do not speak Cantonese, even when the pitch information is non-linguistic or irrelevant.

In order to test the SSCLM hypothesis [1], we used a training task in which participants had to learn new sound categories in the music domain. Participants were trained to associate ascending music intervals with colors, that is, to extract regularities from the intervals and form basic, abstract color-coded categories (this task was adapted from [35]). It was hypothesized that Cantonese-Dutch bilinguals would perform better than their controls, since their SSCLM is more “trained” in learning sound categories in different languages. We also administered a rhythmic pattern category-learning task, as a control for general learning capacities (see [35]).

Lastly, we wanted to test whether Cantonese-Dutch bilinguals will show longer-term language-to-music transfer effects in pitch processing, as previously shown in tone-language speakers [9,10]. We therefore included a set of pitch perception tasks testing different levels of pitch representation. The purpose of these tasks was to detect whether bilinguals’ experience with a tone language and a non-tone language transfers to non-linguistic pitch processing and, if so, determine the level of pitch representation at which this transfer is demonstrated.

## Materials and Methods

### Participants

#### Cantonese-Dutch bilingual participants

The bilingual group comprised 21 Cantonese-Dutch bilingual speakers. From those, two participants were excluded, one on the basis of music experience and the other for failing a hearing screening test. The data from nineteen participants were therefore analysed, including 10 males and 9 females, aged between 17 and 35 years (mean = 24.16, SD = 5.55). Sixteen participants were tested at Radboud University Nijmegen andfive were tested at Leiden University. Recruitment procedures and selection criteria were identical across the two sites. Participants were recruited through advertisements posted on university webpages and through colleagues. Any individual who acquired both languages, Dutch and Cantonese, before the age of 7 was considered an early bilingual. The bilinguals were all raised in Cantonese-speaking environments, as in all cases both their parents were Cantonese speakers. Participants were compensated with 30 euro gift cards.

#### Control participants

Forty-three university students were recruited for the control group from Radboud University’s research participation system database. Participants were all native speakers of Dutch. Since there is no strictly monolingual group in the Netherlands (English is introduced at age 10–12 in all primary schools), the control participants were late bilingual speakers, having learned on average more than two languages in addition to Dutch. Exclusion criteria were 1) failing the hearing screening, 2) being an early bilingual, 3) having more than 3 years of music experience, 4) speaking or learning a tone language or a Dutch tone dialect, and 5) being older than 35 years. A total of eleven participants were excluded for not meeting the criteria: four participants for failing the hearing screening, three for being raised bilingual, one for having music experience, and two for learning and speaking Mandarin or a Limburg tone dialect respectively. The remaining sample consisted of 32 participants, 6 males and 26 females, aged between 18 and 35 years (mean = 22.12, SD = 3.37). One participant did not show up for the second part of the experiment but her data from the first part were used. Participants were rewarded with course credit or 30 euro gift cards.

Due to a measurement error, data from 30 participants in the Vowel-Interval speeded classification task (see below) were rendered unusable. Therefore, an additional control group of native Dutch speakers was recruited for this task. This final group consisted of 22 participants (after excluding one participant for music experience, one for learning a tone language, one for exceeding the age limit, and two for corrupted data). Participants included one male and 21 females, aged between 18 and 24 years (mean = 19.27, SD = 1.98). Participants were rewarded with course credit.

The study was approved by the Ethical Committee of the Radboud University Faculty of Social Sciences, including the testing of 17-year-old bilingual participants and their treatment as adults (including the lack of informed consent from a parent or guardian). All Cantonese-Dutch bilingual and control participants signed an informed consent form prior to participation and had no self-reported neurological or psychiatric disorders.

### Procedure

Testing took place in a sound-proof booth where participants sat comfortably in front of a computer screen. Stimuli were presented and responses were recorded on a Philips computer running an in-house software program[36]. Auditory stimuli were presented at a comfortable intensity level over a pair of Monacor MD-4300 stereo headphones. Response recordings were performed using an IMG Stage line DM- 5000LN Dynamic Microphone. Task order was randomized, with the exception of the Vowel-Interval speeded classification task which for the Cantonese-Dutch bilingual sample was always administered at the beginning of the session so as to match the additional control sample’s conditions. The entire testing procedure took around 200 minutes, split in two sessions of 100 minutes each. Participants had a 10-minute break whenever they desired during the session. Due to time constraints, not all participants completed all the tasks (see Table 1).

**Table 1:** Total Number of Participants that Completed Each Task.

	Cantonese-Dutch bilinguals	Controls
*Task*	N	N
Vowel-Interval Speeded Classification	18	22
Learning to Identify Music Intervals	18	27
Learning to Identify Rhythmic Patterns	18	28
Pitch Change Detection	17	27
Pitch Direction Discrimination	16	28
Simple Tone Sequence	16	32
Transposed Tone Sequence	18	20

### Vowel-Interval Speeded Classification Task

In the vowel-interval speeded classification task, participants heard two disyllabic nonwords (/dalɔ̃/ and /dalø/) differing in their last vowel and sung by a professional French baritone on two intervals, one ascending from the first syllable to the second (F3-F3#), and one descending (F3-A2). They were asked to classify as fast and as accurately as possible the stimuli according to a specified dimension; in the melodic task they were instructed to ignore the words, focus on the melody and classify the intervals as going up or down, and in the phonological task they were instructed to ignore the melody, focus on the words and classify them as “dalo” or “dale”.

There were three conditions in which the two dimensions in the stimuli varied differently: 1) the Redundant condition, when the music interval and phoneme identity varied consistently, which should result in performance gain, 2) the Orthogonal condition, when the variation in the irrelevant dimension was inconsistent, which should cause interference, and 3) the Baseline condition, when the irrelevant dimension was kept constant and only the relevant dimension varied. We measured interference by comparing performance in the orthogonal condition to baseline and gain by comparing performance in the redundant condition to baseline. Response buttons were used and participants’ accuracy and Reaction Times (RTs) were measured. There were three blocks in each task corresponding to three conditions in which the two dimensions in the stimuli varied differently. For a detailed description of stimulus generation procedures see Kolinsky et al. [30] (auditory examples are available online at: http://www.brams.umontreal.ca/plab/research/Stimuli/kolinsky_et_al/index.html).

Participants had a short practice session followed by 72 trials for each block and the whole task took around 30 minutes to complete. Presentation order of tasks and conditions was counterbalanced, and order of trials within conditions was pseudo-randomized. Unlike in Kolinsky et al. [30], there was no time-out beep 2500ms after stimulus presentation. However, we excluded from the analyses RTs larger than 3000ms.

### Learning to Identify Music Intervals

In this task, participants were trained to associate three ascending music intervals, major 2nd (M2), perfect 4th (P4), and perfect 5th (P5), starting from two different reference tones, C4 and F4#, with three arbitrary color labels. The intervals were composed using the Logic Express 9 program (http://www.apple.com/support/logicexpress/) on a MacBook, using piano timbre. The task consisted of a training and a testing phase. Participants were given detailed instructions in which they were introduced to the concept of a pitch interval with emphasis on its relative nature.

In the training phase, participants were presented with a color on a computer screen and were instructed to generate the interval corresponding to the color by trial and error, pressing the space bar for the reference tone and one of three marked keys on the keyboard. Each key corresponded to a specific interval. Feedback was provided, in the form of “correct” or “incorrect” appearing on the screen, so that participants could correctly associate the color label with its matching interval. After the training phase was completed, an identification test followed to assess learning. Participants heard the trained intervals while the three color options appeared on the computer screen. They had to match each interval to its correct color label by clicking on the respective key on a button box corresponding to the color alignment on the screen. The training and testing phases each consisted of 96 trials in random order.

Participants also performed a control task where they had to learn to associate three rhythmic patterns with three color labels. Similarly to Hove et al.’s study [35], all the patterns were in duple meter, consisted of a total of seven tones repeated twice, with one having 1:1 ratio using eighths, the other a 2:1 ratio using triplets, and one with 3:1 ratio using 16ths as distinguishing features. Sequences were presented either in a moderate or in a slow tempo. The patterns were presented in marimba timbre. The concept of a rhythmic pattern was introduced and its relative nature was explained and emphasized during instruction. In the training phase participants were presented with a color on a computer screen and were instructed to generate the rhythmic pattern corresponding to the color by trial and error, by pressing one of three marked keys on a keyboard. Each key corresponded to a specific rhythmic pattern. Feedback was provided, in the form of “correct” or “incorrect” appearing on the screen, in order for participants to correctly identify which color label corresponds to which interval. After training, participants performed an identification test which followed the same structure as the one in the interval condition. Also as in the interval condition, the training and testing phases each consisted of 96 trials and trial presentation was randomized. The entire task took around 40 minutes to complete.

### Pitch Perception Tasks

Four two-alternative forced choice (2AFC) tasks were administered to assess pitch perception. The first two tapped into the initial levels of pitch representation and the remaining two into higher pitch pattern representations [37]. Sine wave tones created with Praat [38] were used for all the pitch perception tasks, following the procedures described by Foxton et al. [37]. The tones included 20ms onset/offset frequency ramps. We used response accuracy as the dependent measure. The inter-stimulus interval was always 1100ms and the inter-trial interval 2000ms long. All tasks were preceded by a short practice session to familiarize participants with the stimuli and procedure.

#### Pitch change detection

Participants were presented with two pairs of sine wave sounds. One pair always consisted of sounds that had the same frequency (500Hz) while the other pair consisted of sounds that differed in frequency (ranging from 490Hz to 510Hz). Each pure tone was 250ms long with a 100ms gap between each pair. The largest difference in frequency was 20Hz and the smallest 2Hz which translates to about 70–17 cents difference in the “different” pair (1 semitone = 100 cents). Participants had to detect which pair was the “different” one by pressing a key on a button box. Half of the time, the different sound [of the pair] was presented first while the trial order was completely randomized for all participants. A total of 80 trials took around seven minutes to complete.

#### Pitch direction discrimination

Participants were presented with two sine wave sounds, one with an upward and the other with a downward glide. Each glide consisted of the initial and final tone, each 250ms long, connected with a linear ramp of 100ms duration. The frequency interval between the initial and final glide frequencies ranged from 4Hz– 50Hz (20 cents– 170 cents, 1 semitone = 100 cents). Participants were asked to indicate which of the two sounds was the one with the upward glide by pressing a button box. The order of upward glide presentation was counterbalanced while the trial order was completely randomized for all participants. A total of 80 trials took around seven minutes to complete.

#### Steady contour pitch sequence task

**Fig 1:**
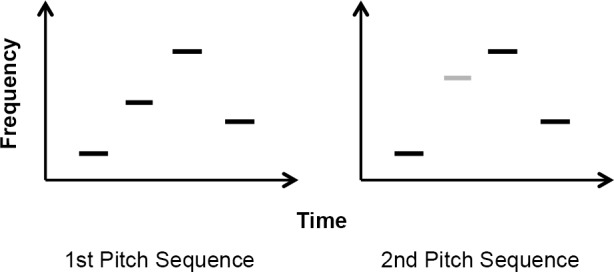
Steady contour pitch sequence task depiction: the second sequence differs from the first in the 2nd tone which has a different frequency but does not violate the contour.

Participants were presented with pairs of four sine wave tone sequences that were either exactly the same or differed in one tone. The difference was such that one tone (either the second or third) in the second sequence was different in frequency from the respective tone (the tone in the same position) in the first sequence. Importantly, the different tone did not violate the melodic contour in the sequence. That is, the direction of the intervals in the two sequences was kept the same (ascending or descending) so that the different tone in the second sequence was different from the first only in terms of absolute frequency (Fig 1). Each tone in the sequence was 250ms long. Participants were asked to report whether the sequences were the same or different by pressing a key on a button box. Half of the pairs were the same and half were different and their presentation order was counterbalanced, while the trial order was completely randomized for all participants. A total of 112 trials took around ten minutes to complete.

#### Steady contour transposed pitch sequence task

This task was identical to the steady contour sequence task with one change: this time the second sequence was always transposed half an octave up or down in frequency. Thus, the second sequence could either have identical frequency intervals as the first or it could contain a different interval that did not violate contour. Participants were asked to ignore the absolute pitch change in the second, transposed, sequence and report whether the sequences were same or different by pressing a key on a button box.

### Control Tasks

Hearing screening was performed with an Oscilla USB-330 audiometer using the random automatic hearing test at 20 dB in 11 frequencies ranging from 125 Hz to 8 kHz in both ears. Since the frequencies used in the experiments were never below 250 Hz or above 4 kHz, participants’ performance in very low and very high frequencies was disregarded. Participants failed the hearing screening when they could not identify frequencies ranging from 250 Hz to 4 kHz at an intensity larger than 30 dB in any of the two ears.

The Raven’s Standard Progressive Matrices test was used to assess general, non-verbal intelligence. Participants’ handedness was assessed using a shortened version of the Edinburgh Handedness Inventory [39] and their short-term memory using the forward digit span adapted from the Dutch version of the Wechsler Adult Intelligence Scale (WAIS).

Background information was collected about the participants’ language and music experience. Participants filled in an online version of the Language history questionnaire for bilingual research [http://www.personal.psu.edu/pul8/questionnaire/L2_questionnaire.html[40]]. They also filled in an in-house questionnaire about their music experience and preferences.

## Results

### Control Measures

The two groups did not differ significantly in age, intelligence (as measured by the Raven’s test of progressive matrices), or music experience (see Table 2). However, they differed significantly in their digit span scores (t = 6.14, p < .001), with the control group having higher scores than the Cantonese-Dutch bilinguals. Despite the discrepancy between the two groups in their digit span scores, it is unlikely that this affected the present results since Digit span did not correlate with any of the measures of interest, apart from accuracy in the control Rhythm Training Identification task for Cantonese-Dutch bilinguals (r = .491, p = .033). Digit span was nevertheless included as a covariate or additional regressor in the primary analyses. As expected, participants differed significantly in the number of languages spoken (t = -3.21, p = .002), with the Cantonese-Dutch bilingual group having learned significantly more languages than the control group.

**Table 2:** Independent T-tests on Demographic Measures Between Groups.

	Cantonese-Dutch bilinguals		Controls		95% CI for Mean Difference		
*Demographics*	M	SD	M	SD		t	df
Age	24.15	5.55	22.12	3.37	-4.53, .472	-1.63	49
Raven’s	55.88	3.80	56.19	3.12	-1.71, 2.32	.304	47
Digit Span	7.00	1.52	9.90	1.69	1.95, 3.85	6.14**	49
Music Experience*	3.31	7.93	5.81	8.88	-2.47, 7.47	1.00	49
Languages	3.61	.697	2.72	1.03	-.886, .276	-3.21**	45

*in months

**p < .01

### Vowel- Interval Speeded Classification Task

#### Phoneme identity discriminability analysis

Since the vowel stimuli were French, we first wanted to test how discriminable they were for our participants. Whereas ø is part of both the Dutch and the Cantonese phoneme inventory, the nasalized ɔ̃ is not. Therefore, we conducted two ANOVAs, one on accuracy scores and one on RTs of the phonological baseline condition, with group as a between-participant factor. The results of the analysis on accuracy did not reveal any effect of phoneme identity [F(1, 39) = 2.19, p>.05] or a main effect of group [F(1, 39) = .832, p>.05]. However, analysis on RTs revealed a significant main effect of phoneme identity [F(1, 39) = 15.42, p < .001, $\eta_{p}^{2}$ = .283], as participants needed more time to respond to ɔ̃ (M = 761ms) compared to ø (M = 728ms, pairwise comparison p < .001, Bonferroni corrected). No other main effect of group or interaction reached significance. Since the two groups did not respond differently to the two phonemes in either speed or accuracy, we assume that vowel discriminability was matched across groups.

#### Order effects

Although task and condition order was counterbalanced between participants, we performed one-way ANOVAs on percentage correct and RTs with order as a between-participant factor. Results did not reveal any effect of task and condition order.

**Fig 2:**
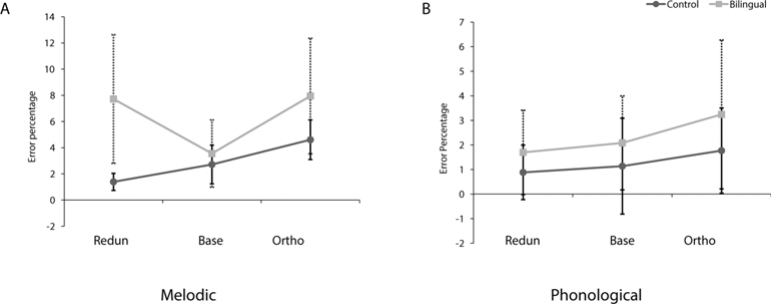
(A) Accuracy scores for the Melodic Interval Speeded Classification task and (B) Accuracy scores for the Phonological Speeded Classification task in the Redundant (Redun), Baseline (Base) and Orthogonal (Ortho) Conditions. Error bars represent standard error of the mean.

Since the dependent measure was categorical (correct/incorrect) rather than continuous, we used mixed logit models for the primary accuracy analysis, based on the aggregate data [41] and using the glmer function provided by the lme4 package in R (version 3.1.2). Generalized linear mixed-effects models with response (1 = correct, 0 = false, logit link function) as a dependent variable tested the effect of Task (Melodic, Phonological), Condition (Baseline, Orthogonal, Redundant), Group (Cantonese-Dutch, control) and their interactions on performance accuracy. Since the two groups differed significantly in their digit span performance, the centered digit span score was added in the model as a covariate. The maximal random effects structure was determined based on convergence by successively removing random effects based on what is justified by the specific experimental design [42]. This maximal term included random slopes for Task and Condition and their interaction for each subject as well as random intercept for subject and item. Since the full model, including the fixed effects, would not converge with this maximal random term, even after correlations between random intercepts and slopes were removed, we simplified it by removing the effect with the smallest SE until the model converged. This left us with the following model: correct ~ 1 + Task * Condition * group + c_digSpan + (Task | subject). The accuracy data are displayed in Fig 2 in terms of percentage correct responses and standards errors.

The analysis revealed a significant effect of Task (*b*_*TaskPhon*_ = .797, *SE* = .400, *p* = .04) where accuracy was significantly higher in the Phonological compared to the Melodic task across groups. There was also a significant effect of Condition with participants performing significantly worse in the Orthogonal condition compared to Baseline (*b*_*ConditionOrth*_ = -.549, *SE* = .198, *p* = .005) and significantly better in the Redundant condition compared to Baseline (*b*_*ConditionRedund*_ = .772, *SE* = .271, *p* = .004). The main effect of Group was not significant (*b*_*Group*_ = -.274, *SE* = .423, *p* = .51) and there was no significant effect of the digit span covariate (*b*_*DigSpan*_ = -.027, *SE* = .071, *p* = .70). Importantly, the Group × Condition and Group × Condition × Task interactions were significant; Cantonese-Dutch participants performed significantly worse in the Redundant condition compared to controls (*b*_*ConditionRedundGroup*_ = -1.635, *SE* = .328, *p* < .001) while they performed significantly better in the Phonological Redundant Condition compared to the Melodic Redundant Condition (*b*_*TaskPhonConditionRedundGroup*_ = 1.648, *SE* = .561, *p* = .003).

#### RT analysis

In all RT analyses, the RTs were estimated by subtracting 750 ms from the RTs measured from the beginning of the stimuli, as the crucial transition between the two notes and vowels was centered at 750 ms after stimulus onset. Only correct responses were analyzed. Any responses given 2500 ms after stimulus presentation were excluded from the analysis.

**Fig 3:**
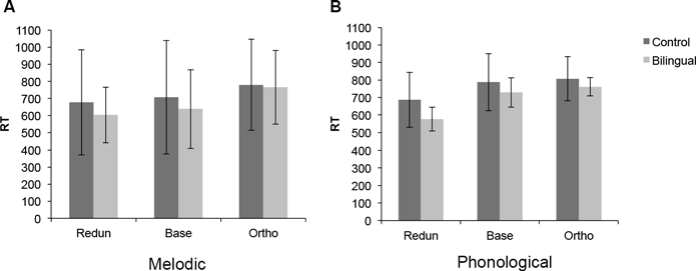
(A) RTs for the Melodic Interval Speeded Classification task and (B) RTs for the Phonological Speeded Classification task in the Redundant (Redun), Baseline (Base) and Orthogonal (Ortho) Conditions. Error bars represent standard error of the mean.

We performed the RT analysis twice: once including all participants and once excluding participants with more errors than two standard deviations above the mean. Since the results remained very similar, we report in full only those based on the whole dataset, as summarized in Fig 3.

Discriminability of the melodic and phonological dimensions was tested with a paired-sample t-test on the baseline condition RTs for both tasks. The comparison did not reveal any significant difference between baseline RTs for the two tasks for either the control [t(23) = -1.435, p = .165] or the Cantonese-Dutch bilingual participants [t(16) = -.859, p = .403].

RTs were analyzed using a 2 (Group) × 2 (Task: Melodic and Phonological) × 3 (Conditions: Baseline, Orthogonal, Redundant) ANOVA. The analysis revealed a significant main effect of condition [F(2, 78) = 60.47, p < .001, $\eta_{p}^{2}$ = .608], as participants were faster in the Redundant condition (M = 645 ms) and slower in the Orthogonal condition (M = 790 ms), compared to Baseline (M = 713 ms, all pairwise comparisons p < .001, Bonferroni corrected) (see Fig 2A and 2B). No main effect of group or task was found (F<1), however, there was a significant task x condition interaction [F(2, 78) = 6.49, p = .002, $\eta_{p}^{2}$ = .143] with slowest RTs for the Orthogonal Melodic condition (M = 806 ms) and fastest for the Redundant Phonological condition (M = 638 ms).

We conducted a separate analysis on gain RTs, calculated by subtracting Redundant RTs from Baseline, and interference RTs, calculated by subtracting Baseline from Orthogonal RTs. The 2 (Group) × 2 (Task) × 2 (Effect: Gain, Interference) ANOVA on RTs revealed a significant Task × Effect interaction [F(39,1) = 16.880, p < .001] with gain in performance being larger in the Phonological task (M = 106ms) compared to the Melodic task (M = 29ms, pairwise comparison p = .005, Bonferroni corrected) and interference larger in the Melodic task (M = 124ms) compared to the Phonological task (M = 30ms, pairwise comparisons p = .001, Bonferroni corrected). There was also a marginal Group effect [F(39, 1) = 2.911, p = .096] which was significant in the trimmed RT dataset [F(28,1) = 4.255, p = .049] and which was driven by overall larger interference and gain effects in the Cantonese-Dutch bilinguals.

In order to make our analyses more comparable to those in Lee & Nusbaum [33], we also performed separate RT analyses for each group. We conducted two separate 2 (Tasks) x 3 (Conditions) repeated measures ANOVAs on RTs. There were different patterns for the control and Cantonese-Dutch bilingual participants. Both groups showed a main effect of condition [Dutch: F(2, 46) = 26.53, p < .001, $\eta_{p}^{2}$ = .536; Cantonese-Dutch bilinguals: F(2, 32) = 33.513, p < .001, partial η^2^ = .677]. However, the task x condition interaction was significant only for Cantonese-Dutch bilinguals [F(2, 32) = 5.57, p = .008, $\eta_{p}^{2}$ = 258]. Cantonese-Dutch bilinguals did not benefit from redundancy in the Melodic condition but showed robust interference effects (p = .002, Bonferroni corrected). In contrast, in the Phonological condition the Cantonese-Dutch bilinguals did not show interference (p = .183, Bonferroni corrected) but did show highly significant redundancy gains (p < .001, Bonferroni corrected).

To summarize, we found a significant three-way interaction between Group, Task and Condition on performance accuracy. The Cantonese-Dutch bilinguals made more errors than the controls in the Redundant condition regardless of task but performed significantly better in the Phonological Redundant condition compared to the Melodic Redundant condition. Although we expected that the Cantonese-Dutch bilingual group would have shorter RTs than controls, there was no main effect of Group in the RT analysis. A separate RT analysis for each group, however, revealed a task × condition interaction only in the Cantonese-Dutch bilinguals, who showed no redundancy gain in the Melodic task and no interference in the Phonological task.

### Learning to Identify Music Intervals

**Fig 4:**
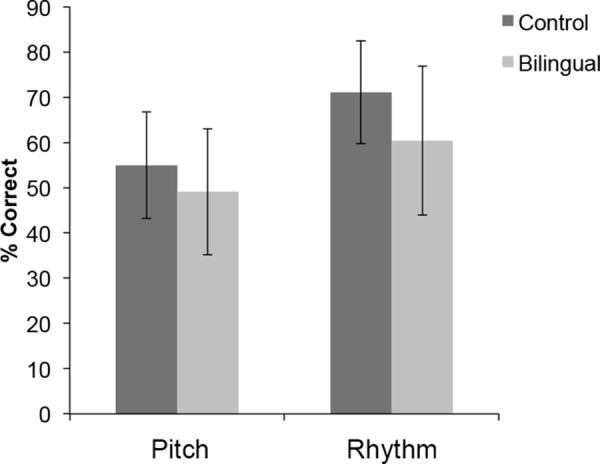
Mean group performance for Pitch Interval and Rhythm Pattern Identification. Error bars represent standard error of the mean.

Overall percentage correct for each task was analyzed using a 2 (group: controls, Cantonese-Dutch bilinguals) x 2 (task: Pitch, Rhythm) ANOVA. Although there was a main effect of task [F(1, 43) = 42.06, p < .001, $\eta_{p}^{2}$ = .494], no task x group interaction was found. Participants overall found the Rhythm task (M = 65.77%) much easier than the Pitch interval task (M = 52.05%). A significant main effect of group [F(1, 43) = 5.96, p = .019, $\eta_{p}^{2}$ = 122] was found, with control (M = 63.05%) participants overall outperforming the Cantonese-Dutch bilinguals (M = 54.77%) (see Fig 4).

We then used generalized linear mixed-effect models with response in the pitch interval task (1 = correct, 0 = false, logit link function) as the dependent variable to test the effect of interval type (M2, P4,P5), reference pitch (C4, F4#), group (Cantonese-Dutch, control) and their interaction terms on performance accuracy. Again, the centered digit span score for each participant was used as a covariate. The maximal model was then reduced in order to reach convergence by successively eliminating the highest order interaction terms and the random terms with the smallest standard error. The non-maximal model which converged included only the main effects [Correct ~ 1 + Interval + Pitch + group + c_digSpan + (Interval | [34] publication bias.onals. ory terval and the rhythm pattern identification learning tasks. subject)]. The random term included random intercepts and slopes for interval type per subject. The results showed a significant effect of interval type with M2 being the easiest and P5 being the most difficult interval to identify (*b*_*IntervalP5*_ = -.317, *SE* = .152, *p* = .03; *b*_*IntervalP4*_ = -.175, *SE* = .126, *p* = .16). There was also a main effect of reference pitch with participants performing better with F4# as the reference pitch (*b*_*PitchF4#*_ = .127, *SE* = .060, *p* = .03). Participants’ digit span score was the most significant predictor of accuracy in the pitch interval learning task (*b*_*DigSpan*_ = .132, *SE* = .048, *p* = .006). There was no significant effect of group (*b*_*Group*_ = -.004, *SE* = .202, *p* = .98).

The same analysis was performed for the rhythm pattern learning task with rhythmic pattern (1:1, 1:2, 1:3), tempo (slow, moderate), group (Cantonese-Dutch, control), their interaction terms and centered digit span as predictors. Since the maximal model did not converge, we followed the successive elimination procedure described above until the model converged. The final model included the main effects without their interactions, random intercepts for subjects and items as well as random slopes for pattern and tempo per subject [Correct ~ 1 + group + Pattern + Tempo + c_digSpan + (Pattern + Tempo | subject) + (1 | TrialID)]. The results revealed a significant effect of digit span (*b*_*DigSpan*_ = .271, *SE* = .099, *p* = .006) but no other predictors reached significance (*b*_*Pattern2*_ = .722, *SE* = .874, *p* = .40;*b*_*Pattern3*_ = .996, *SE* = .890, *p* = .263;*b*_*TempoSlow*_ = - .557, *SE* = .705, *p* = .429;*b*_*Group*_ = .264, *SE* = .421, *p* = .530).

In sum, the pitch identification interval tested whether early experience with a tone and a non-tone language can sharpen sound category learning mechanisms as proposed by the SSCLM hypothesis [1]. Against our expectations, task performance did not reveal a tone/non-tone bilingual advantage in learning new sound categories in our Cantonese-Dutch participants. Performance in the Rhythm task also did not differ between groups which suggests that general associative learning skills were the same across groups.

### Pitch Perception Tasks

**Fig 5:**
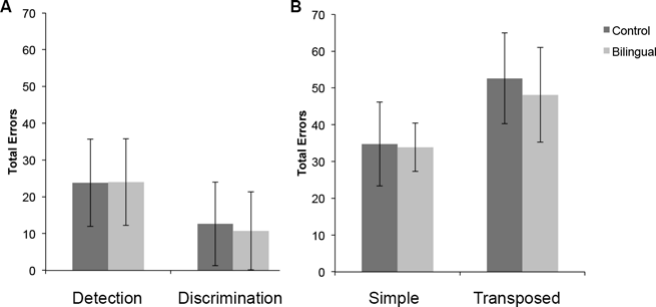
(A) Pitch-change detection and pitch-direction discrimination mean group accuracies. (B) Mean group performance for the steady-contour pitch sequence tasks (simple and transposed). Error bars represent standard error of the mean.

Group differences in pitch perception were tested using Independent-samples t-tests with accuracy as the dependent measure. Overall, there was no difference between the control and the Cantonese-Dutch bilingual groups in any of the pitch perception tasks (see Fig 5. No group difference was found in pitch-change detection accuracy [controls (M = 23.81, SD = 11.85) and Cantonese-Dutch bilinguals (M = 24, SD = 11.74), t(42) = -.051, p > .05]. An item analysis showed that the larger the pitch excursion in the different pair, the fewer the errors participants produced [r = -.897, p < .001], indicating that the task was indeed measuring pitch-change perception sensitivity. We performed a similar item analysis on the pitch-direction discrimination data which showed that the larger the pitch excursion in either pair (upward or downward), the fewer the errors committed by participants [r = -.568, p < .001], as expected. However, no significant group difference was found [controls (M = 12.64, SD = 11.34), Cantonese-Dutch bilinguals (M = 10.12, SD = 10.07), t(42) = .736, p > .05] (see Fig 5A).

Comparisons between control (M = 34.77, SD = 11.41) and Cantonese-Dutch bilingual participants (M = 33.89, SD = 12.35) in the steady contour pitch sequence task were also not significant [t(48) = .256, p > .05]. Likewise, for the Transposed version of this task, no differences were found [controls (M = 52.6, SD = 6.58) and Cantonese-Dutch bilinguals (M = 48.11, SD = 12.87), t(36) = 1.374, p > .05] (see Fig 5B).

The pitch sequence tasks required a Same/Different judgment and, although they have a 2AFC format like the other pitch tasks, they are more susceptible to response biases as they also have characteristics of a reminder task (the second stimulus has to be compared to the first) [43]. We therefore computed and compared Hit rates, False Alarm (FA) rates, and d prime. Results from these measures can be found in Table 3 and are not presented in detail here since participants did not differ significantly in any of them.

**Table 3:** Independent T-tests on the Simple and Transposed Tone Sequence d Prime Measures.

	Cantonese-Dutch bilinguals			Controls			95% CI for Mean Difference		
*d prime*	M	SD	n	M	SD	n		t	df
Simple Tone Sequence	1.152	.880	16	1.285	.772	32	-.366, .631	.536	46
Transposed Tone Sequence	.267	.309	18	.176	.349	20	-.090, -.090	-.843	36

Since the groups differed significantly in their digit span scores, we ran simple linear regressions for the pitch detection, discrimination and sequence (simple and transposed) tasks with Group and Digit Span as the predictors and accuracy and d prime as the dependent variables (separate models per dependent measure). No significant results were found.

To summarize, while previous studies have shown a tone-language advantage in pitch processing, our Cantonese-Dutch bilinguals did not perform better than the controls in a series of pitch detection and discrimination tasks.

## Discussion

In this study we tested the effect of early bilingualism in a tone and a non-tone language on musical pitch perception. Our hypothesis was that early exposure to diverse tonal systems (lexical tone in Cantonese and intonational tones in both Cantonese and Dutch) would sharpen attentional mechanisms involved in tone perception, and hence facilitate the processing of tone in the musical domain. With respect to online speech-music interactions, our results demonstrate that Cantonese-Dutch bilinguals process musical pitch and phonology in a more holistic way than the Dutch control participants. That is, in terms of speed of processing (RTs), Cantonese-Dutch bilinguals showed more interference with varying speech information while performing musical judgments and larger gains when the melodic information varied consistently while performing phonological judgments. In terms of performance accuracy (correct responses), Cantonese-Dutch bilinguals benefited more from consistent variation between melodic and phonological information when performing phonological judgments compared to melodic judgments. The two groups did not differ in their ability to learn new sound categories. We also failed to observe a bilingual tone-language advantage in the pitch-perception tasks.

### Online Processing Interactions between Speech and Music

Previous studies have shown that when speech and music dimensions are processed simultaneously they interact, such that irrelevant variation in one dimension cannot be filtered out and interferes with processing the other dimension, while consistent variation offers performance gains in the form of faster processing [30,44]. Speech and music information are therefore intimately coupled during online processing. Not only do they share resources but they are also perceived integrally.

When asked to perform a task requiring processing of multidimensional stimuli carrying both, melodic and phonological information, our Cantonese-Dutch bilingual participants showed more interference from irrelevant variation in phonology while performing melodic judgments as indicated by their RTs, compared to controls. Using similar multidimensional stimuli, Lee and Nusbaum [33] found that irrelevant variation in segmental information slowed down processing of (non lexical) constant pitch information in Chinese speakers but not in English speakers. Although our Dutch control participants showed interference from phonological variation, the effect was larger for the Cantonese-Dutch participants. Contrary to Lee and Nusbaum, who also found interference from orthogonal steady pitch variations in phonetic performance in Chinese but not English speakers, we found the opposite pattern: the control participants showed interference from music intervals while performing phonological judgments but the Cantonese-Dutch bilinguals did not. This disparity could be due to differences in the pitch stimuli (steady pitch instead of sung music intervals) and/or the segmental stimuli used. In particular, Lee and Nusbaum used syllables that formed actual words in Mandarin, thus carrying semantic in addition to phonological information for Chinese listeners. Caution is therefore warranted when comparing findings across these studies.

In the more comparable Kolinsky et al. [30] study, participants showed asymmetric interference similar to our participants (i.e., more interference in the melodic task). Kolinsky et al. interpret the asymmetry in terms of processing levels, arguing that interval processing (or musical mode of processing) occurs at a later stage compared to phonetic processing and may be more demanding. This could also be the case for the overall asymmetric interference we find in the melodic task.

Interference alone, however, is not enough to argue that two dimensions are processed integrally. Lee and Nusbaum [33] did not include a redundant condition to measure redundancy gains, which according to Garner and Felfoldy [31] are necessary to argue that two dimensions are perceived integrally. Participants in our study showed redundancy gains in both tasks, indicating that the two dimensions, music and speech, were processed holistically. However, the gains were larger for the phonological compared to the melodic task. This asymmetry in gains was more pronounced in the Cantonese-Dutch bilinguals in the RT analysis, and they also had lower error rates in the redundant phonological condition compared to the redundant melodic condition (as indicated by the significant group by task by condition interaction). This is in contrast with the symmetric gains found in Kolinsky et al. [30]. The disparity could be due to the fact that the phonemes used were non-native for our listeners and native for the French listeners in Kolinsky et al. [30]. This might have led to discrepancies in baseline performance in the two studies and subsequently to the asymmetric gains in the present study.

Asymmetric redundancy gains are difficult to interpret solely in the context of stages of processing. If music processing occurs later than phonological processing, its redundant variation should not offer any performance facilitation. According to Garner [45], in the absence of discriminability differences between two dimensions, asymmetric gains or interference can be attributed to selective attention differences acting on top of different levels of perceptual processing. The asymmetric redundancy gain in our data could therefore mean that it is easier for participants to attend selectively to phonological information when fewer processing resources are taken up by redundant melodic variation. The gain is asymmetric because selective attention is less effective when processing melodic information, at least if it is always processed after phonological information.

The asymmetry in gain during the phonological task versus interference in the melodic task was larger for our Cantonese-Dutch bilingual group compared to the control group. We hypothesize that the Cantonese-Dutch bilinguals could not avoid attending to irrelevant phonetic information while processing pitch information because of their linguistic experience. They therefore processed sung words in a more holistic way than control participants. When processing a tone language, attending simultaneously to pitch and phonetic information is essential. Although such simultaneous processing of pitch and speech information should be familiar to Dutch speakers through intonation, pitch variation in that case is usually more dynamic compared to intervals consisting of two music tones. Cantonese, in contrast, includes level tones, which are discriminated in terms of F0 [29]. It is thus plausible that the observed group differences are due to the differences in the participants’ linguistic background.

We did not find evidence for the alternative hypothesis that, being Cantonese-Dutch bilinguals, our participants should have better inhibition and switching abilities, and therefore would show less interference than controls. One could argue that their performance in the phonological task, where they showed no interference but significant redundancy gains, was due to superior attentional control. However, if that were the case, we should have observed the same pattern in the melodic task. Since that was not the case, we favor the interpretation that our results are attributable to the Cantonese-Dutch bilinguals’ tone-language experience, which has shaped the way they integrate phonological and pitch information.

### Learning New Sound Categories

We also tested whether early bilingualism with a tone and a non-tone language has an influence on the ability to learn new sound categories. In particular, we wanted to test the SSCLM hypothesis [1] which suggests that speech and music share the same sound category learning mechanism. Our prediction was that the Cantonese-Dutch bilingual group would be better in learning music interval categories due to a more highly trained, and thus efficient, SSCLM. Contrary to our expectation, we did not observe a bilingual advantage in learning performance. If anything, the control group performed better, although there was no statistically significant difference.

While there is a possibility that the SSCLM hypothesis is not valid, we can speculate about why we could not observe the predicted bilingual advantage. One of the possible reasons is that there was a stronger predictor of performance than experience with a tone and a non-tone language: digit span. Digit span explained significant variance in both the pitch interval and the rhythm pattern identification learning tasks. These learning tasks may therefore be tapping more into short-term memory processes than sound-learning processes. Another reason, perhaps also related to short-term memory abilities, could be the short duration of the training. Participants had to learn to associate three music intervals with three color labels in only 96 trials. This might have been too little exposure to yield any group differences. In Cooper and Wang’s [18] tone training study, for example, no significant differences were found in the first training session but there was an advantage for musicians and tone language speakers over non musicians in the final training session. A second reason for the absence of group effects here could be that participants did not understand the concept of a music interval category. Although special care was taken to explain the music interval as a relative difference between tones, participants probably relied on absolute pitch strategies (given the significant effect of reference pitch), an indication that they failed to grasp the categorical nature of the interval. Again, having multiple learning sessions could have helped participants to understand music intervals and to develop more efficient learning strategies. Furthermore, we cannot exclude the possibility that the Cantonese-Dutch bilinguals’ expertise in intonational and lexical tone might not have been relevant enough to increase the efficiency of the SSCLM in the music domain. Finally, the comparison between the experimental and control groups in the present study was not as large as it could have been. The control participants spoke multiple languages. Thus, while the controls did not speak a tone language, and they were not early bilinguals, any difference in the effect of linguistic experience between them and the Cantonese-Dutch bilinguals was smaller than it would have been if the controls were monolingual speakers. Future studies (e.g., with more training sessions and with a strictly monolingual control group) are needed in order to draw any firm conclusions about the SSCLM hypothesis.

### Pitch Perception

Finally, we also tested Cantonese-Dutch bilinguals’ pitch-perception abilities by administering four pitch-perception tasks. Each task aimed at different levels of pitch representation [37] ranging from simple pitch-change detection and pitch-direction discrimination to pitch sequence processing. Despite tone language benefits documented previously in the literature, in this study we failed to find an advantage of pitch perception in bilingual speakers of a tone and non-tone language. Bilinguals and controls did not differ in their response accuracy in any of the levels of pitch representation.

Again, caution should be taken in interpreting these results. Participants performed tasks on pure sine wave tones with which they probably did not have any prior experience, in comparison to acoustically richer musical or lexical tones. Furthermore, the majority of pitch excursions in the pitch change detection and pitch direction discrimination were smaller than a semitone. The smallest difference in Cantonese level tones is on the order of a semitone [29].Given that the language-experience advantage decreases as the pitch excursions tested become smaller than those occurring naturally in the respective tone language spoken[9,11], we could speculate that this was the reason we did not find group differences.

In addition, the tone sequences in the sequence task did not have any melodic structure and thus lacked contextual information that could have aided participants (e.g. dominant key information). As a previous study comparing Cantonese to English speakers has shown, music pitch perception benefits of tone language speakers are limited to the integration of musical tones over a larger melodic context [10]. The absence of such a context may have posed significant challenges, especially in the transposed version of the pitch sequence task, as revealed from accuracy scores which were at chance level for both groups. The fact that changes were never contour violations added to the task difficulty, perhaps obscuring group differences that could have arisen under other conditions.

Another reason why there was no difference between groups in the pitch tasks could be, as noted with respect to the learning task, the contrast between the experimental and control groups was smaller than it could have been. Furthermore, our Cantonese-Dutch bilingual participants were living in the Netherlands and thus were immersed in a Dutch-speaking environment instead of a Cantonese one. In their study, Bidelman and colleagues [9] found a positive correlation between the amount of exposure to Cantonese tones and perceptual pitch advantages in their Cantonese participants. This could also explain why our Cantonese-Dutch participants did not outperform their Dutch peers in pitch perception tasks.

The effect sizes for speech-music transfer effects reported in tone-language speakers are very small even in studies with large sample sizes (e.g. [10]). It thus seems reasonable to conclude that, if they exist, the effects of speaking a tone and a non-tone language on musical pitch processing are not large enough to be detected in our tasks. This in turn suggests that there may be limitations to the role that experience can play in shaping pitch processing abilities. Furthermore, individual differences in pitch processing aptitude might influence performance by interacting with experience, and/or those differences may tend to be larger than those due to experience. Lastly, the fact that the transfer effects are difficult to detect indicates that although the two domains cannot be completely modular, some aspects of music pitch processing may be relatively impenetrable to the effects of linguistic experience. Further research with more power, a better control group, and more sensitive behavioral measures could nevertheless reveal effects on musical pitch processing stemming from bilingual experience with pitch.

## Conclusions

We tested whether early experience with a tone language and a non-tone language (in a group of early Cantonese-Dutch bilinguals) has effects on music processing abilities. We found no bilingual advantage in learning music interval categories or in pitch perception tasks. Especially in the context of the ongoing debate about the strength of the evidence for a bilingual advantage in executive function (compare [46] and [47]), it should be clear that these null results are certainly not the last word on whether early bilinguals have an advantage in the ability to learn new sound categories or to process musical pitch. But we did find evidence for more holistic processing of sung stimuli in the Cantonese-Dutch bilinguals compared to the Dutch controls. This is, to the best of our knowledge, the first study to show that being bilingual in a tone language and a non-tone language influences online processing interactions between music and speech. Not only did the Cantonese-Dutch bilinguals find it more difficult to filter our irrelevant phonological information while judging melodic intervals, but they also showed greater performance gains when melodic information varied consistently with phonological information. Although greater pitch interference in tone-language speakers has been demonstrated before [33], this is the first time that redundancy gains, crucial for the integrality argument [31], have also been shown in tone-language speakers. We interpret these results as arising from the bilinguals’ experience with their tone language, where pitch and segmental information are integral properties of speech segments and spoken words. This is fundamentally different from the case of non-tone languages, where pitch is primarily an independent feature added on top of segmental information. The necessity of processing segmental and suprasegmental information integrally in a tone language thus appears to transfer to situations where speech and music are processed jointly.
